# Application of Polyvinyl Alcohol–Ethylene Glycol Hydrogel Technology for Removing Animal Glue in Book Restoration Based on Fluorescent Labeling Evaluation

**DOI:** 10.3390/nano14231878

**Published:** 2024-11-22

**Authors:** Jia Wang, Yuting Xu, Canxin Tian, Yunjiang Yu, Changwei Zou

**Affiliations:** 1 Library, Lingnan Normal University, Zhanjiang 524048, China; wangjia@lingnan.edu.cn; 2 Department of Physics, Lingnan Normal University, Zhanjiang 524048, China; 13433129012@163.com (Y.X.); cxtian@lingnan.edu.cn (C.T.); sidui@lingnan.edu.cn (Y.Y.)

**Keywords:** PVA-EG hydrogel, animal glue removal, book restoration, fluorescence labeling

## Abstract

This study developed a novel material based on polyvinyl alcohol–ethylene glycol (PVA-EG) hydrogel and systematically evaluated its potential application in the removal of animal glue from book surfaces. The microstructure, surface properties, and mechanical characteristics of the PVA-EG hydrogel were analyzed using X-ray diffraction (XRD), Fourier-transform infrared spectroscopy (FTIR), contact angle measurements, a universal testing machine, and a dynamic mechanical analysis (DMA). The introduction of ethylene glycol (EG) could weaken hydrogen bonding interactions between PVA chains to enhance the molecular chain flexibility of the hydrogel. Notably, the 10% PVA-EG hydrogel shows better crystallinity, higher hydrophilicity, and optimal balance between mechanical strength and flexibility compared to pure PVA, which is conducive to improving the efficiency of the removal of animal glue. Additionally, the effectiveness of the process of removing animal glue was verified by real-time monitoring using europium nitrate at a concentration of 0.4% (*w*/*v*) as a fluorescent marker. Such hydrogels with high mechanical properties, strong surface hydrophilicity, good removal efficiency, and gentle treatment characteristics have potential applications in the restoration of cultural heritage.

## 1. Introduction

The protection of cultural heritage is of great significance to the inheritance of human civilization. Many precious ancient books, such as ancient texts, calligraphy, and paintings, and paper artifacts like murals, have developed problems of aging and deterioration over time due to the use of traditional adhesives like animal glue [1,2,3]. The animal glue becomes brittle over a long period, leading to damage to paper fibers and pigment layers, which poses significant challenges to the restoration of ancient books [4,5,6]. Traditional cleaning methods like mechanical scraping and the use of organic solvents can cause additional damage to ancient books and lack precise control over the cleaning process [7]. These methods may damage the substrate materials of ancient books or leave harmful residues, affecting their long-term preservation. There is an urgent need to develop a gentle, controllable, and non-destructive method of removing animal glue for the restoration of classical books.

In recent years, the new hydrogel cleaning system has gradually become an essential tool for the cleaning of ancient books due to its controllability and gentleness [8,9,10]. These hydrogels can confine cleaning agents within the gel network, achieving controllable release of the cleaning solution, and avoiding the risk of the penetration and diffusion of liquid cleaning agents, thereby reducing the damage to ancient books.

Polyvinyl alcohol (PVA)-based hydrogels have been widely used in the field of ancient book cleaning due to their biocompatibility, tunable mechanical properties, and good adhesiveness [11,12,13,14]. By blending PVA hydrogels with other natural or synthetic polymers—such as polyvinylpyrrolidone (PVP), starch, chitosan, agarose, methacrylates, etc.—or by introducing cross-linking agents, their properties can be further enhanced, including increased mechanical strength, water retention capacity, and plasticity [15,16,17,18,19,20]. Recent research has further broadened the applications of PVA hydrogels in cleaning ancient books. For example, the “green” PVA/starch cryogels prepared by introducing starch not only have excellent cleaning performance but also enhance ecological compatibility [21]. In addition, PVA-based sponge-like cryogels fabricated through freeze–thaw cycles and solvent exchange methods possess adjustable pore structures and excellent diffusion properties, which make them ideal for cleaning complex surfaces [22,23,24]. The optimized semi-interpenetrating p(HEMA)/PVP hydrogel accelerates the gel preparation process and enhances the effectiveness of cleaning artwork surfaces [25].

To tackle the challenge of removing animal glue, recent studies proposed the use of a heat-induced PVA hydrogel dissolution method to effectively eliminate animal glue from ancient mural surfaces [26]. This method avoids damage of the mural substrate base by partially dissolving the PVA hydrogel under heat to control the release of hot water, gradually dissolving and removing the animal glue layer. Moreover, microemulsion-loaded PVA/PEI hydrogels have also been used to clean animal glue and dirt from the surfaces of murals and ancient coins, demonstrating their potential in cleaning ancient books [27]. In addition to PVA-based hydrogels, other types of gel materials have also been used in cultural heritage cleaning. For example, the reusable cross-linked gels based on poly(ethyl methacrylate) (PEMA) with adjustable flexibility and affinity for organic solvents can be used to clean aged coatings on artwork surfaces [28]. The combination of bioenzymes and biosurfactants has been used to remove mold spots from paper artifacts, showing good cleaning effectiveness and material compatibility [29]. Polyelectrolyte hydrogels have been used for non-destructive cleaning of murals, enhancing the mechanical properties of the gels and their adsorption capacity for dyes and metal ions by introducing chitosan and charged copolymers [30]. More research is now focusing on green chemistry and renewable materials to enhance the sustainability and eco-friendliness of cleaning methods [31,32]. For instance, nanostructured bio-based organogels derived from castor oil and prepared via a sustainable polyurethane cross-linking method can be used for cleaning water-sensitive artworks [33]. Starch nanoparticles prepared through non-solvent methods using green solvents can be used to reinforce fragile painting layers [34].

Current hydrogels still present some specific limitations in practical applications, including restricted adaptability to complex surfaces, complex preparation procedures, and the possibility of gel residue [35,36,37,38,39,40]. To solve these problems, this study focuses on developing an optimized PVA-EG hydrogel by adjusting PVA concentrations (6%, 8%, 10%, 12%) and incorporating ethylene glycol (EG) as an additive to achieve hydrogels with enhanced mechanical properties and water absorption capacity. We prepared the hydrogel by the low-temperature freeze–thaw cycling and solvent replacement methods. The structure and properties of the hydrogels were characterized by XRD, FTIR, contact angle measurements, mechanical testing, and DMA. The results indicated that introducing EG facilitated the orderly arrangement of PVA molecular chains, enhancing the crystallinity and mechanical strength of the hydrogels. Fluorescently labeled animal glue was used to simulate ancient books, and the cleaning effectiveness of PVA-EG hydrogels was evaluated on glue layers of different thicknesses and we confirmed the feasibility of this approach. This optimizing method offers a new gentle, efficient, and controllable technique for cultural heritage preservation.

## 2. Materials and Methods

### 2.1. Materials

Polyvinyl alcohol (PVA, 96.0–98.0% hydrolysis degree) and ethylene glycol (EG, analytical grade) were used directly in the experiments without further purification. Fluorescent dye (europium nitrate solution) was used to simulate the book surface and to prepare fluorescent animal glue.

### 2.2. Preparation of PVA-EG Hydrogel

PVA-EG hydrogels were prepared by the low-temperature freeze–thaw cycling method and solvent exchange technique. First, PVA particles of different concentrations (6%, 8%, 10%, 12%) were dissolved in a mixture of deionized water and ethylene glycol (mass ratio of 4:6), and stirred continuously at 90 °C until the PVA was fully dissolved. The solution was then poured into Petri dishes and frozen at −24 °C for 24 h, followed by thawing at room temperature for 4 h. To guarantee the formation of a stable hydrogel structure, the freeze–thaw process was repeated three times. A 10% PVA hydrogel was prepared following the same method. After the freeze–thaw cycles, the PVA-EG hydrogel was immersed in deionized water for 24 h, changing the water every 3 h to replace the EG.

### 2.3. Characterization Techniques

The crystalline structure of PVA hydrogel samples was analyzed using an X-ray diffractometer (Empyrean, PANalytical, Almelo, The Netherlands). The testing range was from 10° to 80° (2θ) with a scanning speed of 2°/min, using a Cu K_α_ radiation source (λ = 1.5406 Å). The FTIR spectra of the hydrogel samples were recorded using a Fourier-transform infrared spectrometer (IRPrestige-21, Shimadzu, Kyoto, Japan) over a scanning range of 3750–750 cm^−1^, with an attenuated total reflectance (ATR) accessory. The ATR accessory used a zinc selenide (ZnSe) crystal to ensure good contact with the sample surface and obtain high-quality spectral data. The surface wettability and hydrophilicity of PVA hydrogel samples with different concentrations were tested using a contact angle measuring instrument (DSA100, Krüss GmbH, Hamburg, Germany). Each hydrogel sample was tested three times to ensure the repeatability of the measurements, thereby evaluating the surface wettability and hydrophilicity of the samples. Tensile tests of the PVA hydrogel samples were conducted using a universal testing machine (Instron 3365, Instron Corporation, Boston, MA, USA) in accordance with ASTM D638 standards. The sample dimensions were 12 mm in width, 50 mm in length, and 5 mm in thickness, and the tensile test loading speed was set to 5 mm/min. Each sample was tested three times, and the average values were recorded. The viscoelastic properties of the PVA hydrogel samples were tested in the shear mode using a dynamic mechanical analyzer (DMA 242 E Artemis, Netzsch, Stuttgart, Germany). The test conditions included a frequency of 1 Hz and a temperature range from 30 °C to 50 °C, with a heating rate of 2 °C/min.

### 2.4. Fluorescent Animal Glue Removal Experiment

#### 2.4.1. Preparation of Fluorescent Animal Glue

To simulate the animal glue layers found in book restoration and to determine the optimal concentration of a fluorescent marker, experiments were conducted to evaluate the effect of various concentrations of europium nitrate (Eu(NO_3_)_3_) on fluorescence intensity. In total, 10 g of animal glue granules was added to 90 mL of deionized water and heated to 60 °C in a water bath with continuous stirring until fully dissolved. Then, the europium nitrate solution was added to the animal glue solution to prepare 0.1%, 0.2%, 0.4%, 0.6%, 0.8%, and 1.0% (*w*/*v*) europium nitrate solutions. The fluorescence spectra of the europium nitrate animal glue solutions at different concentrations were measured using a fluorescence spectrophotometer. In this study, fluorescence measurements were conducted using a steady-state and transient fluorescence spectrometer (FLS1000, Edinburgh Instruments, Livingston, UK), with an excitation wavelength set at 318 nm and an emission scanning range of 550–750 nm.

#### 2.4.2. Preparation of Simulated Samples

The prepared europium nitrate fluorescent animal glue solution was evenly coated onto 5 cm × 5 cm filter paper with thicknesses of 5 μm, 10 μm, 20 μm, and 50 μm by using a blade coater. After coating, the samples were dried at 25 °C in a constant-temperature and -humidity chamber for 24 h to ensure full curing of the glue layer.

#### 2.4.3. Animal Glue Removal Experiment on Simulated Samples

PVA hydrogels were cut into 5 cm × 5 cm sheets with a thickness of 5 mm and placed over the fluorescent animal glue layer. An infrared heating lamp (150 W) was placed about 10 cm above the hydrogel, and the temperature was controlled at 45 °C. The removal time was adjusted according to the thickness of the glue layer. At each removal time point, the fluorescence spectrum of the sample was measured with a fluorescence spectrophotometer, and the removal efficiency was assessed based on changes in fluorescence intensity.

#### 2.4.4. Animal Glue Removal Experiment on Book Samples

To verify the feasibility of PVA hydrogels in real-world book restoration, we selected a page adhered with animal glue for the experiment. The thickness of the animal glue layer on the book page was measured using a high-precision thickness gauge, and the appropriate removal method was selected based on the thickness. The method followed the fluorescent animal glue removal experiment. At each time point, the surface of the book page was measured using a Fourier-transform infrared spectrometer (FTIR) to evaluate the removal effectiveness.

## 3. Results and Discussion

### 3.1. Crystal Structure Analysis

**Figure 1:**
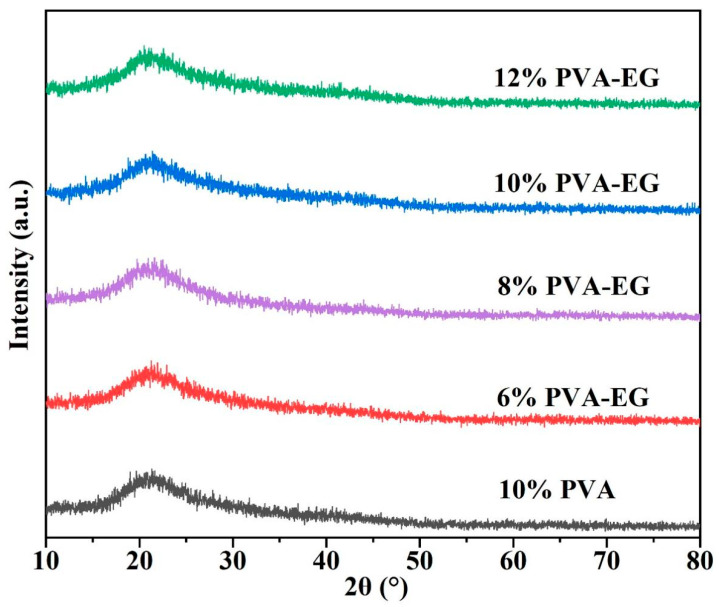
XRD patterns of PVA-EG hydrogel samples at various concentrations.

Figure 1 shows the changes in diffraction peaks of PVA-EG hydrogel samples with different concentrations. All samples exhibited a distinct diffraction peak at around 19.4°, corresponding to the (101) crystal plane of PVA, indicating that the introduction of EG did not disrupt the crystal structure of PVA [41]. In contrast, the addition of EG effectively promoted the orderly arrangement of PVA segments, further improving the crystalline structure of the samples. Notably, in the 10% and 12% PVA-EG samples, the intensity and sharpness of the diffraction peaks were significantly enhanced compared to other samples. This can be attributed to the formation of additional hydrogen bonds due to the presence of EG, which strengthened the intermolecular interactions between PVA chains, thereby promoting a more ordered molecular arrangement.

### 3.2. Chemical Structure Characterization

**Figure 2:**
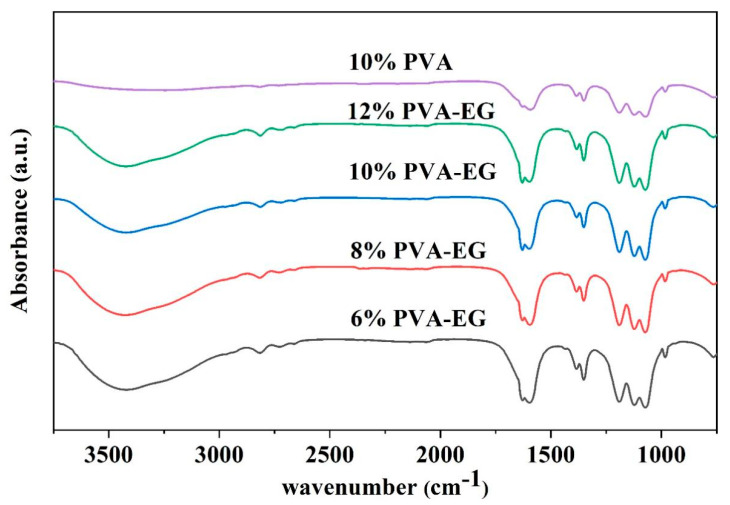
FTIR analysis of PVA-EG hydrogels at different concentrations.

Figure 2 illustrates the changes in FTIR absorption peaks of PVA-EG hydrogel samples at various concentrations. The results show that the O-H stretching vibration peak (3300 cm^−1^) was gradually broadened and the intensity was decreased as the concentration of EG increased. This suggests that the addition of EG can weaken the hydrogen bonding interactions between PVA molecular chains by inserting itself between the PVA segments. The O-H peak in the pure PVA sample is more distinct, indicating a relatively dense hydrogen bond network [42]. In samples with higher PVA content, more PVA molecular chains can form hydrogen bonds with EG molecules, which partially inhibits the complete hydrolysis of PVA and retains the acetyl (-O-C=O) groups; however, the change in the C=O signal was not significant [43]. Additionally, changes in the absorption peak were observed around 1700 cm^−1^. According to the study of Tretinnikov, the carbonyl absorption peak in PVA can reflect changes in molecular arrangement. One of the peaks in the doublet may correspond to the carbonyl groups in PVA molecules, while the other may result from interactions between EG and PVA, possibly forming a new hydrogen bond network and leading to a more ordered arrangement of PVA segments [42].

### 3.3. Surface Wettability Analysis

**Figure 3:**
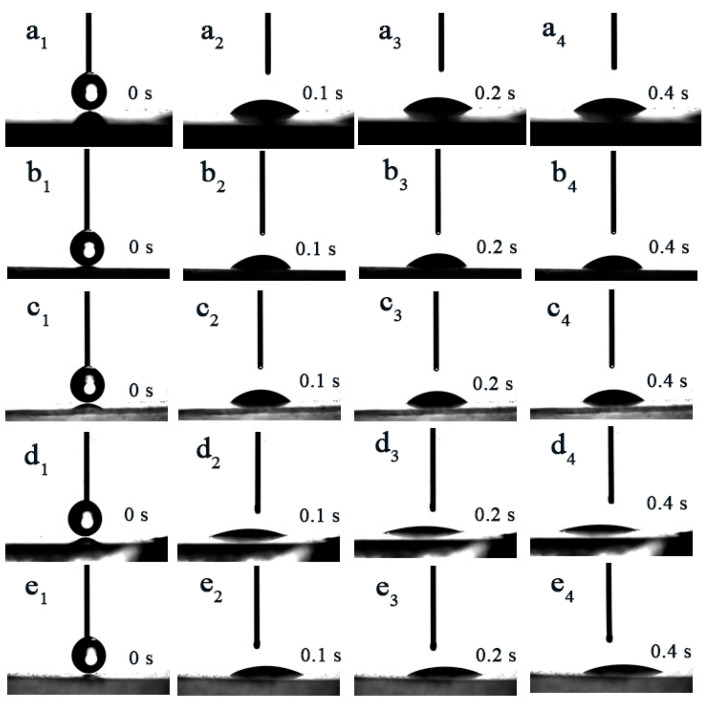
Contact angle measurement results of PVA-EG hydrogel samples at different concentrations: (**a**) 10% pure PVA hydrogel, (**b**) 6% PVA-EG hydrogel, (**c**) 8% PVA-EG hydrogel, (**d**) 10% PVA-EG hydrogel, (**e**) 12% PVA-EG hydrogel.

Figure 3 illustrates the changes in the wettability of PVA-EG hydrogels at various concentrations. The pure PVA sample had a larger initial contact angle, and the contact angle decreased slowly over time, indicating poor surface wettability and relatively weak water absorption capacity. This could be attributed to the stronger hydrogen bonding between pure PVA molecular chains, causing tighter molecular packing and less exposure of hydrophilic groups, thus reducing interaction with water molecules. With the increase in the EG, the contact angle of the hydrogel gradually decreased, and the wettability significantly improved. Particularly in the 10% and 12% PVA-EG samples, the water droplets spread significantly at 0.1 s and were almost fully absorbed after 0.4 s with the contact angle approaching 0. This indicates that the introduction of EG significantly improved the hydrophilicity and water absorption capacity of the PVA hydrogel. EG molecules formed new hydrogen bonds with PVA molecular chains, increasing the polarity of the hydrogel and the density of hydrophilic groups. This change in structure increased the surface free energy of the hydrogel, facilitating interactions with water molecules and accelerating water absorption and penetration. During heating, the hydrogel with excellent water absorption can release more moisture, maintaining the glue layer in a hydrated state and further improving the removal efficiency.

### 3.4. Mechanical Property Testing

**Figure 4:**
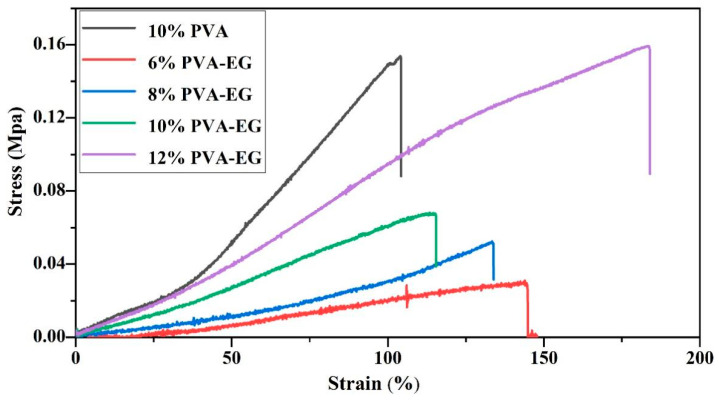
Stress–strain curves of PVA-EG hydrogels at different concentrations.

Figure 4 illustrates the stress–strain curves of PVA-EG hydrogels with various concentrations. The results indicate that the mechanical properties of the hydrogel significantly improve with the addition of EG, particularly in samples with higher PVA concentrations. Specifically, the 6% PVA-EG hydrogel has good flexibility with a maximum strain of about 150%, but its mechanical strength is low with a maximum stress of only 0.05 ± 0.003 MPa, limiting its use in applications requiring mechanical support. The 8% PVA-EG hydrogel strikes a good balance between rigidity and flexibility, with a maximum strain of 120% and a maximum stress of 0.08 ± 0.005 MPa, showing significant improvement over the 6% concentration. For the 10% PVA-EG hydrogel, its mechanical strength was notably improved, reaching a maximum stress of 0.12 ± 0.009 MPa while maintaining a maximum strain of around 110%. This balance between strength and ductility allows it to effectively tackle stubborn animal glue without causing excessive stress concentration on the book surface. The 12% PVA-EG hydrogel exhibited the strongest mechanical strength, with a maximum stress approaching 0.15 ± 0.011 MPa and a strain of 150%, demonstrating excellent strength and ductility. However, its relatively low flexibility could pose a risk of damaging fragile surfaces when working with delicate substrates. The 10% PVA-EG hydrogel demonstrated the best mechanical properties, providing sufficient strength to remove stubborn animal glue while maintaining moderate flexibility, effectively meeting various complex book restoration needs.

### 3.5. Dynamic Mechanical Analysis (DMA)

**Figure 5:**
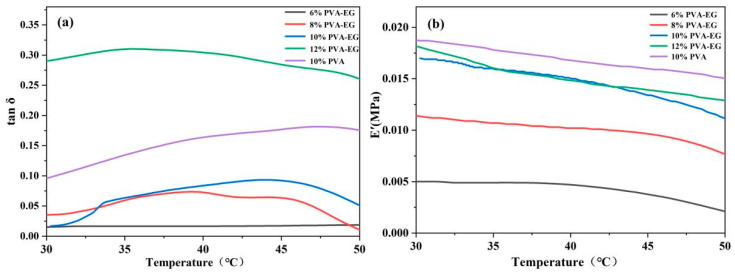
Viscoelastic behavior of PVA-EG hydrogels at different concentrations in the temperature range of 30 °C to 50 °C. (**a**) Loss factor tan δ of PVA-EG hydrogels; (**b**) Storage modulus E’ of PVA-EG hydrogels.

Figure 5 shows the viscoelastic behavior of PVA-EG hydrogels at different concentrations in the temperature range of 30 °C to 50 °C. The 6% PVA-EG hydrogel exhibited the lowest E’ value across the entire temperature range, especially when the temperature exceeded 45 °C, where E’ dropped to 0.003 ± 0.0001 MPa, indicating lower elasticity and insufficient structural rigidity. In contrast, the E’ value of the 8% PVA-EG hydrogel was 0.009 ± 0.0004 MPa at 45 °C, indicating the elasticity with slight improvement compared to the 6% PVA-EG. The tan δ value was 0.05, suggesting a better balance with viscoelasticity, but the elasticity at high temperatures was still low, making it suitable for more moderate removal applications. The 10% PVA-EG hydrogel exhibited a relatively high and stable E’ value from 30 °C to 50 °C with an E’ value of 0.012 ± 0.0005 MPa at 45 °C, indicating excellent elasticity and mechanical performance. The tan δ value remained below 0.10, indicating a good balance between viscosity and elasticity with minimal energy dissipation. The E’ value of the 12% PVA-EG hydrogel was 0.013 ± 0.0005 MPa at 45 °C, showing relatively good elasticity, but its tan δ value reached 0.30, indicating a significant increase in the viscous component and energy dissipation, which may lead to a decrease in mechanical performance during high-temperature operations. The 10% PVA-EG hydrogel achieved an optimal balance between the storage modulus and loss factor, combining high elasticity with low energy dissipation, making it suitable for applications requiring structural stability and mechanical performance under high-temperature conditions, especially in the removal of animal glue.

### 3.6. Analysis of Fluorescent Animal Glue Removaling Results

#### 3.6.1. Optimization of Europium Nitrate Concentration and Its Effect on Fluorescent Labeling

**Figure 6:**
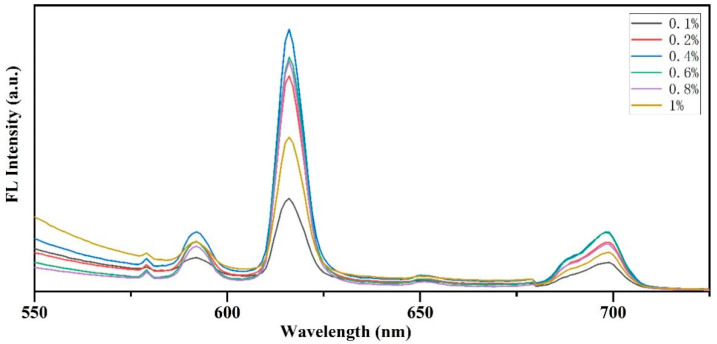
Effect of different europium nitrate concentrations on fluorescence intensity of animal glue.

Figure 6 illustrates the impact of various europium nitrate concentrations on the fluorescence intensity of animal glue. The results show that the fluorescence intensity first increased and then decreased with the increase in europium nitrate concentration. The fluorescence intensity peaked at a concentration of 0.4%. When the concentration exceeded 0.4%, the fluorescence intensity dropped significantly, indicating a concentration quenching effect. The position of the fluorescence emission peak remained stable at all concentrations, indicating that the concentration changes primarily affected fluorescence intensity, not emission wavelength. Therefore, 0.4% (*w*/*v*) europium nitrate was selected as the optimal concentration for fluorescent labeling, providing a strong and stable fluorescence signal for subsequent experiments.

#### 3.6.2. Removal Efficiency of Simulated Animal Glue Layers by PVA-EG Hydrogel

**Figure 7:**
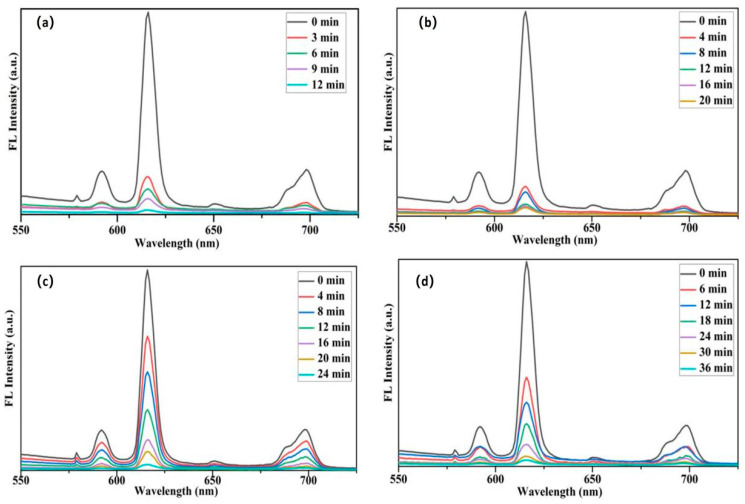
Removal efficiency of PVA-EG hydrogel on fluorescent-labeled animal glue layers of different thicknesses. (**a**) Fluorescence intensity changes for the 5 μm glue layer; (**b**) Fluorescence intensity changes for the 10 μm glue layer; (**c**) Fluorescence intensity changes for the 20 μm glue layer; (**d**) Fluorescence intensity changes for the 50 μm glue layer.

Figure 7 shows the removal efficiency of PVA-EG hydrogel on fluorescent-labeled animal glue of different thicknesses. The results show that the fluorescence intensity rapidly decreased for thin glue layers (5 and 10 μm), reaching baseline levels within 12 min and 20 min, respectively, indicating that the glue layers were effectively removed. This indicates that the water in the hydrogel can quickly penetrate, soften, and dissolve the thin glue layers. For medium-thickness glue layers (20 μm), the fluorescence intensity significantly decreased within 24 min, and the fluorescence signal was almost eliminated, suggesting that most of the animal glue had been removed. For thicker glue layers (50 μm), the fluorescence intensity dropped significantly, nearing baseline levels after 36 min of treatment, indicating that most of the animal glue had been removed. These experiments demonstrate that the PVA-EG hydrogel is effective in removing animal glue layers of various thicknesses with the removal efficiency inversely proportional to the thickness of the glue layer.

#### 3.6.3. Application of PVA-EG Hydrogel in Removal of Animal Glue from Book Pages

**Figure 8:**
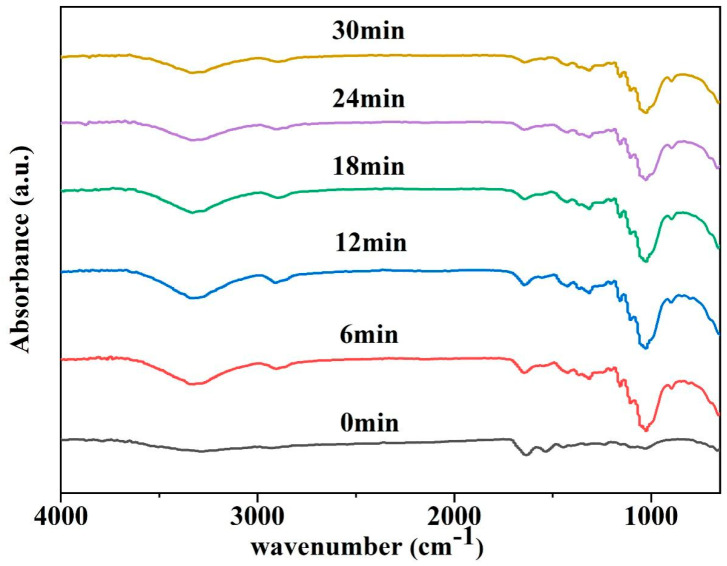
The effect of PVA-EG hydrogel on the removal of animal glue from book pages.

To assess the practical effectiveness of the PVA-EG hydrogel, we applied it to remove a 35 μm thick animal glue layer from the pages of a book. During the removal process, we used FTIR to monitor the change in the glue layer, focusing on the characteristic absorption peaks of the animal glue—amide I (1650 cm^−1^) and amide II (1550 cm^−1^). Figure 8 illustrates the effectiveness of the PVA-EG hydrogel in removing animal glue from book pages. The results showed that the intensity of the amide peak gradually weakened and disappeared with the increase in treatment time. After 12 min of treatment, the intensity of the amide peak decreased significantly, indicating that part of the adhesive layer had been removed. At 24 min, the amide peak disappeared almost completely, indicating that most of the animal glue had been removed. After 30 min, the amide peak had completely disappeared, and the infrared spectrum matched that of the untreated paper substrate, confirming that the adhesive layer had been fully removed. These results demonstrate that PVA-EG hydrogels can efficiently remove animal glue from book pages within a relatively short time, and the removal efficiency improves with increasing treatment time.

#### 3.6.4. Mechanism of Animal Glue Removal by PVA-EG Hydrogel

From a theoretical perspective, the mechanism of efficient animal glue removal by PVA-EG hydrogel can be attributed to the synergistic effect of its molecular structure and interfacial physicochemical properties. The addition of EG plays a key role in the hydrogel system by regulating hydrogen bonding between PVA molecular chains, which significantly improves the crystallinity and mechanical properties of the material. EG enhances the flexibility of PVA molecular chains by forming new hydrogen bonds and enables the hydrogel to conform to complex surfaces, ensuring close coverage of the animal glue surface during application. The addition of EG also significantly improved the hydrophilicity of the hydrogel, enhancing its water transfer capacity. This characteristic allows water to quickly penetrate the animal glue layer, promoting its hydrolysis and softening. The mechanical stability of the hydrogel structure was strengthened by the regulation of EG, enabling the PVA-EG hydrogel to maintain prolonged effective contact during operation, ensuring the continuity and thoroughness of the removal process.

## 4. Conclusions

This study employed various methods, including XRD, FTIR, contact angle measurements, a universal tensile testing machine, and DMA, to explore the potential application of PVA-EG hydrogels in gently removing animal glue from book surfaces. The addition of EG improved both the crystallinity and mechanical properties of the hydrogel by modulating hydrogen bonding interactions among PVA molecular chains. The XRD analysis revealed that as crystallinity increased, the crystal structure of PVA-EG hydrogels became more organized. Notably, the 10% PVA-EG hydrogel achieved an optimal balance between mechanical strength and flexibility. The FTIR analysis further illustrated that EG facilitated rearrangement and flexibility within the molecular chains, thereby enhancing both mechanical stability and removal efficiency of the hydrogel. A combined analysis using DMA tests and stress–strain curves demonstrated that PVA-EG hydrogels possess excellent energy storage capacity and viscoelastic balance across varying temperatures. Specifically, the 10% PVA-EG hydrogel maintained stable elasticity and strength even at elevated temperatures while also exhibiting commendable extensibility with low energy dissipation. Overall, our results suggest that the PVA-EG hydrogel can effectively yet gently remove glue layers in a relatively short time frame. The introduction of EG into this formulation successfully achieved a harmonious balance among removal efficiency, mechanical performance, and viscoelasticity.

## Data Availability

Data are contained within the article.
